# Removal of the Lower Jaw, for Osteo-Sarcoma of Immense Size

**Published:** 1860-12

**Authors:** Geo. C. Blackman

**Affiliations:** Professor of Surgery in the Medical College of Ohio; Surgeon to the Commercial St. John’s and St. Mary’s Hospitals, Cincinnati; Fellow of the Royal Medical and Chirurgical Society of London.


### REMOVAL OF THE LOWER JAW, FOR OSTEO-SARCOMA OF IMMENSE SIZE.

BY GEO. C. BLACKMAN, M.D.

Professor of Surgery in the Medical College of Ohio; Surgeon to the Commercial St. John's and St. Mary's Hospitals, Cincinnati; Fellow of the Royal Medical and Chirurgical Society of London.

On the 2d of July, 1859, Lemuel Hinedon, a negro, aged 30, was admitted into St. John's Hospital, for the removal of the lower jaw, which was affected throughout a considerable extent with the disease known as osteo-sarcoma. The magnitude of the tumor caused him to present a frightful aspect. From the history of the case as recorded by Dr. John A. Billings, then resident physician at St. John's Hospital, it appears that nine years before, one of the molar teeth on the right side of the lower jaw became loose, and was somewhat painful. Soon after, he noticed a small tumor on the bone, which, however, gave him no uneasiness; it increased slowly, but steadily, up to the time of his admission. In some parts the tumor was quite hard, in others it had an elastic feel, imparting even the sensation of indistinct fluctuation. Deglutition and respiration not seriously disturbed, although the power of mastication was nearly lost. On the buccal aspect of the tumor were two small ulcerated patches, through which, he stated, from time to time he had lost large quantities of blood. Just previous to his admission his strength had been reduced by an alarming attack of hemorrhage. The appearance of the patient is well represented in plate I.

The patient having been brought under the influence of chloroform, with the assistance of Drs. Trippler, Foster, Fries and Muscroft, I commenced by making a single curvilinear incision, commencing in front of the ear, on the right side, and passing over the most prominent portion of the tumor, to near the left angle of the jaw. The soft parts were next rapidly dissected from the bone. Both facial arteries were divided, and were found to be much enlarged. The hemorrhage from the left was controlled by pressure, but the retraction of the vessels beneath the huge mass on the right side rendered this ineffectual; and this, together with the gushing from every part of the diseased bone, caused the patient to lose an enormous quantity of blood. Seizing the most prominent part of the tumor with both hands, I wrenched out the largest portion, and the persulphate of iron was freely applied to the bleeding surfaces. The flow was at length checked, but not until the pulse and respiration had become almost imperceptible. In a few moments he rallied, when I proceeded to remove the body of the jaw as far back as the angle. At this point no trace of osseous structure remained. The exhaustion of the patient now became extreme, and it was evident he could not then survive the completion of the operation. Beef-tea and whisky were administered, both by the mouth and rectum. Aided by artificial respiration, reaction was established in the course of half an hour, when the wound was closed, and the patient comfortable in bed. Hemorrhage occurred during the night, but was controlled without difficulty. On the third day the stitches were removed, and the wound was almost entirely united.

He still felt considerably prostrated. On the sixth day, however, he was able to leave his bed, and to walk around the room. Two weeks after the operation he felt as well as ever.

On the of 7th August, thirty-six days after the first operation, I proceeded to remove the remaining portion of the tumor. Finding that the cheek hung loose and flabby, I made two curvilinear incisions, inclosing a flap of skin about four inches in width. The neck and condyle of the bone were healthy; but the ramus, with the overlapping structures, was so degenerated and blended that it was impossible to distinguish it. The morbid mass extended deeply towards the root of the tongue. After the division of the integuments, the knife was laid aside; and with the bone gouge forceps I succeeded in breaking up the mass completely, and in extirpating to the articulation. The large mass involving the root of the tongue was raised by an assistant, so that I succeeded with less difficulty than I had anticipated in wrenching out the entire morbid structure. The hemorrhage was readily controlled by the application of persulphate of iron, as fast as fresh portions were exposed by the gouge forceps.

Nothing of particular moment occurred during the patient's convalescence, which was rapid. On the 28th of August he went to work, his health being perfectly reëstablished. He could masticate with ease. Even after the removal of the flap above mentioned, for some weeks, the cheek appeared rather loose, and the motor powers of the right side of the face were greatly impaired, as the portio dura was so intermingled with the degenerated mass that it was necessarily divided, and portions of it removed with the tumor. The parotid gland itself was indeed from the same reason in greater part extirpated. We think, however, that any one, after examining fig. 3, from a photograph taken eight weeks after the first operation, will agree with us that the appearance of his face is very satisfactory. In July last, a year having passed since he came under our treatment, Lemuel was carefully examined by Dr. Foster and myself, and we could discover no signs of a return of the disease. It may be well to state, in this connection, that there is reason to believe that he has not gone to bed sober a single night since he recovered from the last operation.

There are some points of interest in the above case, to which we desire to call special attention. In the first place, ought not the surgeon, before attempting the removal of so large a tumor of the lower jaw, to ligate the primitive carotid artery? In support of this practice, as is well known, we have the high authority of Dr. Valentine Mott, whose name is so honorably associated with the early history of these operations for osteo-sarcoma.

In the *American Journal of the Medical Sciences* for October, 1856, we published the report of a case in which, for a most formidable osteo-sarcomatous tumor, we removed the *entire* lower jaw. The tumor had been growing for forty years, and had obtained such fearful magnitude that the patient was threatened with suffocation. In that operation, although the facial arteries bled freely, they were readily controlled by pressure until the ligatures were applied. In other cases, where we had removed the lower jaw from the articulation, we had encountered no serious difficulty in arresting the hemorrhage. At the close of our report, to which we have referred, after alluding to other instances in which tumors of this kind, of immense size, had been successfully removed without resorting to the ligature of the carotid, we used the following language: "I will only add, that if in the terrible operation performed by Prof. Syme, as well as by myself, but a few ounces of blood were lost, surely, in operations of less magnitude in this region the ligature of the primitive carotid must be unnecessary." Now, does my case of the negro Lemuel call for a modification of the above opinion? Let us briefly analyze the cases reported by Dr. Mott. We quote from our edition of Mott's Velpeau, vol. ii., p. 347. In his first case, Catharine Bucklero, the ligature of the carotid caused her to become "agitated and perturbed to a great degree." This "remarkable agitation" led to the postponement of the operation, which was performed the next day. In reference to the hemorrhage we find the following:

"Very little blood was lost during this operation. Two arteries only of any size were divided, the facial and lingual, and these only required the ligatures to the branch extremities; but each end was tied for safety. Another small artery behind, and a little underneath the posterior angle of the jaw, yielded some blood and was tied." The tumor in this case was of moderate size; yet we find, after the ligature of the carotid, that five ligatures were applied to divided vessels during the operation.

In Dr. Mott's second case (op. cit., p. 354,) the tumor was much larger, presenting "an appearance in size equal to that of his head." The carotid was tied, and *four* ligatures were applied during the removal of the jaw. The operation was performed at noon, on the 15th of May, 1823, and he died on the 19th at 4 o'clock P. M. The post-mortem showed extensive thoracic disease; "each lung exhibited marks of high inflammation throughout their whole extent;" and within the pericardium was a pint of yellow serum. There was also "a massy deposit" of coagulable lymph in the anterior mediastinum. We shall have occasion again to refer specially to the cause of death in this instance.

In Dr. Mott's third case (op. cit., p. 357) the tumor had been of rapid growth, and was about three inches in its transverse, and from five to six in its longitudinal diameter. The primitive carotid was tied, and yet the report states "the hemorrhage was exceedingly profuse," requiring, we are told, the ligature of some fifteen to twenty vessels."

I am not aware of any other cases reported in detail by Dr. Mott, although Velpeau, in his Table of Cases of Exsection of the Lower Jaw, affixes to his name nine cases—two disarticulations and three deaths. In each of the cases to which we have referred, Dr. Mott removed the bone at the articulation. Dr. Mott is of the opinion that there will be less hemorrhage if the removal of the jaw is performed the day after the ligature of the carotid, as the branches of that vessel will have more time to contract. Now, at page 345, op. cit., we find that M. V. De Lavacherie, of Leige, Belgium, lost a patient from hemorrhage immediately after the operation, although the carotid had been tied the day before. In February, 1848, we disarticulated the lower jaw, where the face was greatly swollen, presenting even a fungoid appearance. The hemorrhage from the facial artery ceased without a ligature; and even that from the trunk, or one of the main branches, of the internal maxilliary (caused by an unexpected plunge of the patient), was readily controlled by pressing a piece of sponge into the wound. On the 25th of March, 1848, we disarticulated the left half of the lower jaw, removing the bone (osteo-sarcoma) from the chin to the articulation. Only one vessel, a branch from the internal maxilliary, was tied during the whole operation. In our case of disarticulation on both sides,—removal of the entire bone for osteo-sarcoma—(*Am. Jour. Med. Sciencs*, October, 1856) not more than eight ounces of blood were lost; and in another case where we removed, for necrosis, at the articulation, the hemorrhage was trifling. It was not until we encountered our fifth case of disarticulation—and we had had several operations where the body of the bone was only removed—that our patient had a narrow escape from death by hemorrhage.

Altogether, we have had *five* cases of disarticulation of the lower jaw, a number equal to that of any European, and exceeding that of any American surgeon mentioned in Velpeau's statistics, op. cit., p. 339, vol. ii. In these and in five others in which we have removed the body of the bone, we have never tied the carotid artery as a preliminary step. Our only fatal case is that where we removed the entire bone; and we believe the numerous physicians who witnessed that operation attributed, with myself, the death to the conjoined depressing influences of chloroform and intense heat, the thermometer ranging during the greater part of the day at 100° Fahr.

In the case of the negro on whom Dr. Mott operated, it may, we think, fairly be a question whether the patient did not die in consequence of the ligature of the carotid. The appearances presented at the autopsy were precisely those mentioned by Mr. James Miller, in a paper published in the *London and Edinburg Monthly Journal of Medical Sciences*, for January, 1842, the object of which was to show that inflammation of the lungs is the most common cause of death after the ligature of the main arteries of the neck. Indeed, Dr. Mott himself in 1820 called the attention of the profession to the fact that the ligature of the primitive carotid may aggravate *existing* pulmonary disease, as was the case in which he resorted to this step to lessen the flow of blood to a fungoid tumor of the neck which he was about to extirpate (*N. Y. Hosp. Med. and Surg. Reg.*, 1820.) The same result followed one of our own operations, in June, 1843, undertaken for the purpose of arresting the growth of a large bleeding encephaloid tumor of the neck. Besides the pulmonary and cerebral difficulties which have been clearly proved to follow the ligature of the primitive carotid artery, there is another fearful risk from the operation. Mr. Crisp in his Treatise on the diseases of the Blood-Vessels (London, 1847), has given us the details of twenty-one cases in which the carotid was tied for aneurism, and of the eleven fatal cases—ten only having been successful—five died from *hemorrhage!*

The statistics collected by Dr. Norris and published in the *American Journal of Medical Sciences*, for July, 1847, show the serious character of the operation of ligating the primitive carotid artery; for of one hundred and forty-nine cases, thirty-two were fatal, from hemorrhage, cerebral or pulmonary disease. Here, then, we have one death in four and seven-tenths cases, from an operation recommended to us by the very highest authority, as a *precautionary* step in the removal of tumors of the lower jaw. The mortality is about equal to that from the latter operation itself—one in four—of one hundred and sixty cases collected by Velpeau.

From all the facts to which we have referred, we feel authorized to reject the ligature of the primitive carotid, as practiced by Dr. Mott, in the exsection of the lower jaw. We expect in a very few weeks to operate in another case, in which the tumor (osteo-sarcoma) is fully as large as that which forms the subject of the present paper; and we have no idea of applying a ligature to the primitive carotid. Compression, persulphate of iron, and ligatures to divided vessels, are all the means which we expect to employ to guard against hemorrhage.

The case of the negro Lemuel presents another point worthy of notice. Mr. Fergusson, in his Practical Surgery (Lond. ed., 1857, p. 668), has given us the following rule in reference to the removal of integument: "Whatever bulk the tumor may be in any part of the bone, the whole of the skin should always be retained; for it will soon contract, however much it may be distended." With all due deference to the opinion of this accomplished surgeon, we maintain that the results in the above case lend no support to the rule inculcated; and even after a year from the time of the operation, notwithstanding our liberal removal of integument, a little more contraction seemed desirable. However, as before stated, we think no one who saw Lemuel before the operation can find fault with his present appearance.

Again, it will be remembered that in our second operation we used the bone gouge forceps to break up and remove the morbid mass. Our experience with this instrument in similar operations leads us to speak of it with unqualified praise. A few months since we removed with it the upper jaw of a lady affected with osteo-sarcoma, the face, of course, afterwards not being in the least disfigured. The tumor presented precisely the same appearance as that represented in Mr. Fergusson's work, fig. 357, and for the removal of which Mr. F. made an incision in the mesial line in the hollow of the lip. With the bone gouge forceps in similar cases, and even of much larger dimensions, no external incision is necessary.

Finally, in the case of Lemuel we required two operations to complete the extirpation of the enormous mass. No one ever questioned the boldness or skill of Dieffenbach; and with all his skill and daring, he once thought it expedient in a similar case to resort to three different operations. A graphic description of this case may be found in the chapter on exsection of the lower jaw, in his work on Operative Surgery. The difficulties encountered in some of these tumors must deter any prudent surgeon from the attempt to complete the task at a single operation. In our own case we were well satisfied to bring the patient safely through, even at the second trial.

NOTE.—In the above report, when discussing the propriety or necessity of ligating the primitive caroted as a preliminary step in the exsection of the lower jaw, I stated that I expected soon to operate in a case of almost equal magnitude, and that I should not apply a ligature to the caroted. On the 12th inst. I removed the bone from the articulation on the left to the first molar on the right side, and although the tumor was of enormous size, I had no trouble from hemorrhage, the facial artery alone requiring a ligature. The patient was a female, eighteen years of age, and the tumor was of long duration, but had been increasing very rapidly of late. The exsection was completed in six minutes, and although there was no trouble from retraction of the tongue—which was secured by a cord—there was at times considerable difficulty of breathing, and the patient died in five hours after the operation. A mixture of one part of chloroform and two of sulphuric ether was used as an anæsthetic, and previous to its administration stimulus was given. Just as I was completing the disarticulation a large vein was divided, and I heard a gurgling noise, such as has been described as indicating the admission of air. The collapse following this was instantaneous, and from it the patient never rallied. The autopsy revealed nothing to shed any light as to the cause of death.

Cincinnati, Nov. 16, 1860. G. C. B.

